# Zonation of mangrove flora and fauna in a subtropical estuarine wetland based on surface elevation

**DOI:** 10.1002/ece3.6467

**Published:** 2020-06-04

**Authors:** Wei Ma, Wenqing Wang, Chaoyi Tang, Guogui Chen, Mao Wang

**Affiliations:** ^1^ Key Laboratory of the Coastal and Wetland Ecosystems (Xiamen University) Ministry of Education College of the Environment & Ecology Xiamen University Xiamen China

**Keywords:** crab, mangrove, mollusc, sea‐level rise, species zonation, surface elevation

## Abstract

In the context of sea‐level rise (SLR), an understanding of the spatial distributions of mangrove flora and fauna is required for effective ecosystem management and conservation. These distributions are greatly affected by tidal inundation, and surface elevation is a reliable quantitative indicator of the effects of tidal inundation. Most recent studies have focused exclusively on the quantitative relationships between mangrove‐plant zonation and surface elevation, neglecting mangrove fauna. Here, we measured surface elevation along six transects through the mangrove forests of a subtropical estuarine wetland in Zhenzhu Bay (Guangxi, China), using a real‐time kinematic global positioning system. We identified the mangrove plants along each transect and investigated the spatial distributions of arboreal, epifaunal, and infaunal molluscs, as well as infaunal crabs, using traditional quadrats. Our results indicated that almost all mangrove forests in the bay were distributed within the 400–750 m intertidal zone, between the local mean sea level and mean high water (119 cm above mean sea level). Mangrove plants exhibited obvious zonation patterns, and different species tended to inhabit different niches along the elevation gradient: *Aegiceras corniculatum* dominated in seaward locations while *Lumnitzera racemosa* dominated in landward areas. Mangrove molluscs also showed distinct patterns of spatial zonation related to surface elevation, independent of life‐form and season. The spatial distributions of some molluscs were correlated to the relative abundances of certain mangrove plants. In contrast, the spatial distributions of crabs were not related to surface elevation. To the best of our knowledge, this is the first study to explicitly quantify the influences of surface elevation on the spatial distributions of mangrove fauna. This characterization of the vertical ranges of various flora and fauna in mangrove forests provides a basic framework for future studies aimed at predicting changes in the structure and functions of mangrove forests in response to SLR.

## Graphical abstract

The spatial distributions of mangrove plants and molluscs showed obvious patterns of zonation correlating with surface elevation. Mangrove flora and fauna within each habitat niche occupied distinct vertical ranges along the elevation gradient, irrespective of season. This is the first quantitative study to explicitly examine the influences of surface elevation on the spatial distributions of mangrove fauna within the intertidal zone.

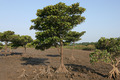

## 1 INTRODUCTION

**FIGURE 1:**
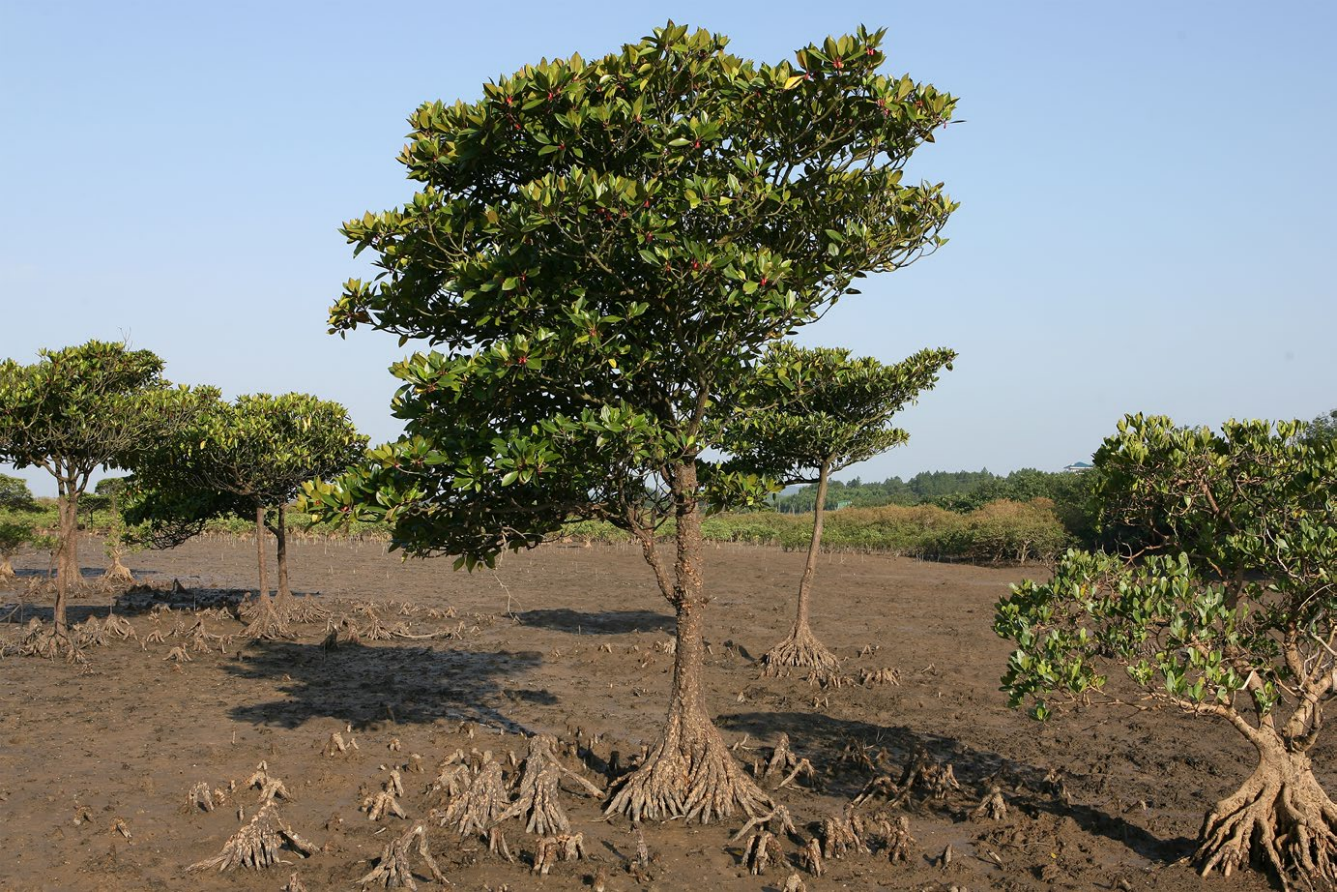
Mangrove plant (*Bruguiera gymnorhiza*) in Zhenzhu Bay, China. Photographer: Bo Su

Mangrove forests are typically distributed in the intertidal region, between the mean sea level (MSL) and the highest spring tide, in tropical and subtropical coastal regions worldwide (Alongi, 2009; Figure 1). In mangrove ecosystems, species zonation is ubiquitous, and the multiple environmental gradients that give rise to this phenomenon have been described in dozens of individual mangrove swamps over the past century (Ball, 1988a; He, Lai, Fan, Wang, & Zheng, 2007; Watson, 1928). Tidal inundation drives the vertical distributions of mangrove plants (Crase, Liedloff, Vesk, Burgman, & Wintle, 2013; Leong, Friess, Crase, Lee, & Webb, 2018) and is an ideal proxy for other environmental drivers that affect plant growth, including salinity, soil texture, and redox potential (Ellison, Mukherjee, & Karim, 2000). The degree of tidal inundation, which is largely regulated by local geomorphology (Thom, 1967), can be reliably and cost‐effectively estimated based on surface elevation (Leong et al., 2018). Most previous studies of zonation patterns in mangrove forests have been qualitative descriptions of the distribution patterns of mangrove plants in the intertidal zone (Chapman, 1976; Watson, 1928). However, several recent studies have attempted to quantify the relationships between surface elevation and mangrove plant distributions (Fu, Zhang, Ao, Wang, & Wang, 2019; Leong et al., 2018; Zhu, Hou, Weng, & Chen, 2019).

The mangrove forest is a unique ecological environment with a distinct fauna (Kathiresan & Bingham, 2001). Molluscs and crabs are the most abundant mangrove macroinvertebrates (Macnae, 1968). Similar to the floral zonation patterns observed in mangrove forests, macroinvertebrate zonation patterns are complex and affected by the frequency of tidal inundation (Alongi, 2009; Martins, 2001; Ragionieri, Fratini, & Cannicci, 2015; Reid, 1985). At present, mangrove forests are threatened by increasing tidal inundation due to sea‐level rise (SLR) (Ellison, 1993; Lovelock et al., 2015; Traill et al., 2011). To predict possible changes in species distributions in response to rising sea levels, it is important to assess how the zonation patterns of mangrove flora and fauna change in response to surface elevation (Di Nitto et al., 2014). Unfortunately, quantitative data describing the distributions of mangrove fauna with respect to elevation in mangrove forests are sparse.

With the development of unmanned airborne vehicles and remote sensing technologies such as LiDAR, highly accurate measurements of surface elevation are now possible in mangrove forests (Crase et al., 2013). However, accurate and robust assessments of mangrove structure at the level of individual trees are difficult (Wang, Jia, Yin, & Tian, 2019), because available algorithms are hindered by the high clumping densities of the mangrove trees and by the limited variations in height among neighboring trees (Heenkenda, Joyce, & Maier, 2015; Yin & Wang, 2019). Indeed, ground‐based surface elevation surveys, performed using highly accurate instruments that account for complex microtopography, are preferable for the estimation of inundation patterns and species distributions in mangrove forests (Ellison et al., 2000; Fu et al., 2019; Leong et al., 2018). Individual mangrove tree measurements made during such surveys are also highly accurate, because these measurements are not obstructed by external factors. Ground‐based surface elevation surveys are suitable for studies that include several mangrove transects and that require traditional quadrats for flora and fauna assessments (Ellison et al., 2000).

In this study, we used a highly accurate ground‐based surveying method to quantify the relationship between species distributions and surface elevation in a mangrove community in southwestern China. The main goal of this study was to quantitatively evaluate how surface elevation governed the spatial distributions of mangrove flora and fauna. We first determined whether mangrove flora and fauna clustered in recognizable vertical zones corresponding to surface elevation to confirm whether there were vertical zonation patterns in mangrove flora and fauna. We then calculated the expected mean elevations of several mangrove species (six plants, 29 molluscs, and four crabs), taking into account seasonal changes, to compare how different species responded to different elevations.

## 2 METHODS

### 2.1 Study site

**FIGURE 2:**
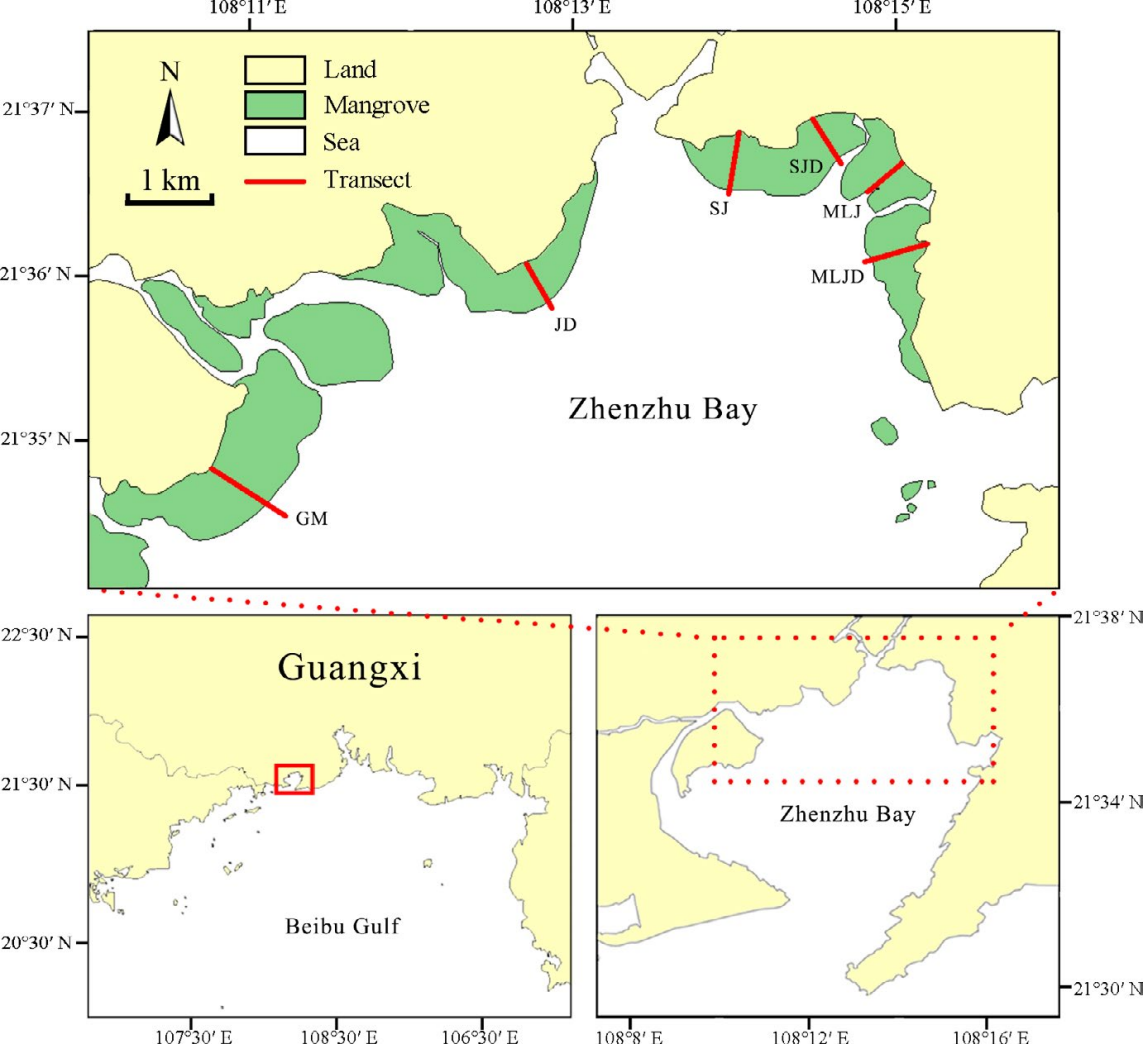
Location of the six transects (red lines) through the mangrove forests in Zhenzhu Bay, Guangxi, China. Each transect runs from the seaward forest edge to the landward forest edge. GM, JD, SJ, SJD, MLJ, and MLJD correspond to the local names of the mangrove forests

This study was conducted at Zhenzhu Bay (21°29′–21°38′N, 108°08′–108°17′E), in Beilun Estuary National Nature Reserve, Guangxi, China. This area was designated a Ramsar site in 2008 (Figure 2). Zhenzhu Bay is a sheltered, funnel‐shaped bay, with an area of about 94 km^2^, that includes 17.33 km^2^ of mangrove forests. At the top of the bay are the outlets of the Jiangping and Huangzhu Rivers. The bay is subject to diurnal tides, with a mean tidal range of 2.24 m and a spring tidal range of 5.05 m; the mean high water (MHW) is 1.19 m above MSL. The average annual salinity is 29.10. The bay has a subtropical monsoon climate. The annual average temperature is 22.5°C, the average temperature in July (the hottest month) is 28.6°C, and the average temperature in January (the coldest month) is 14.1°C. The annual precipitation is 2,220 mm, with most rain falling in the summer (rainy season), and the least rain falling in the winter (dry season) (EBCBS, 1993). The most abundant mangrove plants in the bay are *Aegiceras corniculatum*, *Bruguiera gymnorhiza*, *Kandelia obovata*, and *Avicennia marina*, with the relative abundance of each species varying with surface elevation.

### 2.2 Topographical field survey and sampling

Six transects (each approximately 400–750 m long) were established across the five main mangrove forests (Figure 2), from the seaward forest edge to the shore. In order to fully investigate the mangrove forests of the bay, we ensured that the transects covered a broad geographical area: from the bay mouth to the top of the bay. All transects were at least 1 km apart. Elevation along each transect was measured in 5–10 m surface intervals that were relatively flat, using a Global Navigation Satellite System‐Real Time Kinematic GPS unit (G970 GNSS RTK, UniStrong Inc.). This unit has a vertical precision of 15 mm. The elevation of each surface interval was converted to the Chinese Height Datum using point correction, performed based on control points located 3 km from the study area. The elevation of the local MSL relative to the Chinese Height Datum was 0.34 m (EBCBS, 1993). Therefore, surface elevation relative to the local MSL was determined by subtracting 0.34 m from the measured elevation. Next, we established sampling sites at 25 cm vertical increments along each transect from the seaward edge to the landward edge. Because the elevation range varied among transects, the number of sampling sites along each transect also differed. In addition, due to topographic fluctuations, some transects included more than one site at the same height.

**FIGURE 3:**
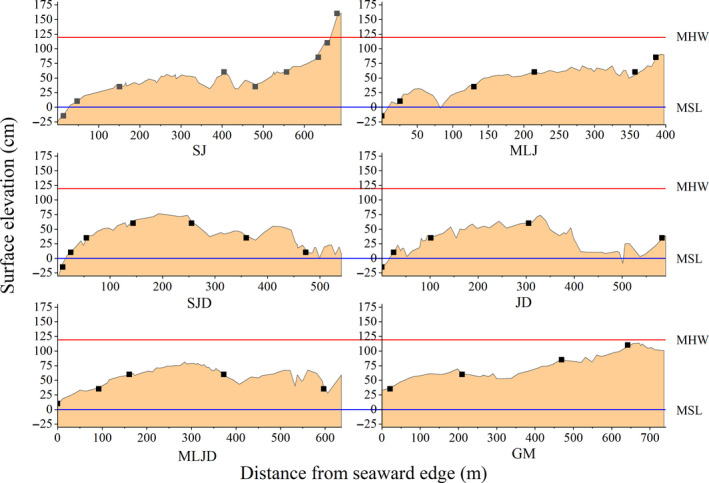
The vertical profiles of the six transects (in centimeters above mean sea level). The blue and red lines indicate the mean sea level (MSL) line and the mean high water (MHW) line, respectively. The locations of the sampling sites within each transect are indicated using the symbol ■. GM, JD, SJ, SJD, MLJ, and MLJD correspond to the local names of the mangrove forests

Surface elevations along the mangrove transects were non‐linear; we typically observed a small rise and fall in elevation between the shore and the seaward edge (Figure 3). Across all six transects, elevation ranged from −34.91 to 155.47 cm above MSL, and 97.3% of the mangrove forests were distributed within the intertidal zone between the MSL and the MHW (Figure 3). We established 36 sampling sites at 25 cm vertical intervals between −15 and 150 cm above MSL (Figure 3). There was no sampling site at 135 cm because there was a gap in the mangrove forest at this elevation.

At each sampling site, because there was only one layer in the vegetation canopy, mangrove plant composition and abundance were recorded using the number of individuals (for individuals >0.5 m tall) in three randomly selected quadrats (5 × 5 m; 10 m apart). We sampled the arboreal, epifaunal, and infaunal molluscan communities, as well as the infaunal crab communities, at each site in July 2017 (rainy season) and in January 2018 (dry season). To collect arboreal molluscs, all specimens attached to trunks, leaves, prop roots, and other tree parts were collected by hand in each mangrove quadrat. To collect epifaunal molluscs, we randomly designated 5 epifaunal‐mollusc quadrats (1 m × 1 m; 5 m apart) per sampling site, and all molluscs on the sediment surfaces of these quadrats were collected. To collect infaunal molluscs and crabs, we randomly designated a 25 × 25 cm area in each epifaunal‐mollusc quadrat; we then sieved the uppermost 30 cm of the sediment in these areas through a 1‐mm mesh (Liu, Wang, Wang, Fu, & Lu, 2016). All specimens were identified to species following Okutani (Okutani, 2000) and Wang, Zhang, Ma, Cai, and Zhang (2016), and then counted and weighted. Five soil samples per sampling site were collected randomly, and soil salinity was determined based on conductivity (following Bao, 2000).

### 2.3 Data analysis

Cluster analyses were used to determine whether our flora and fauna data (mangrove plants, molluscs, and crabs) indicated zonation along the elevation gradient. In this technique, we used a triangular matrix based on the Bray‐Curtis similarity index of the 4th‐root‐transformed species density data from each sampling site to reduce the effects of the more abundant taxa. We then analyzed the data using the group average clustering method, in which similarity profiles (SIMPROF) indicate whether clusters represent patterns of community structure that differ significantly (*p* < .05) from random spatial structures; this method also determines the number of assemblages in each cluster (Clarke, Somerfield, & Gorley, 2008). Similarity percentage analyses (SIMPERs) were used to identify the species that segregated into different communities in the cluster analysis. All analyses were performed using PRIMER v6.0 (Clarke & Gorley, 2006).

Randomization tests developed for niche overlap models were also used to test for floral and faunal zonation patterns along the elevation gradient (following Ellison et al., 2000). We calculated niche overlap using Pianka's overlap index (Pianka, 1973), based on individual numbers of species at each sampling site with niche breadth retained and reshuffled zero states. The mean and variance of Pianka's index of overlap were both compared with a null model generated using Randomization Algorithm 3 (RA3), based on 1,000 random samplings of the abundance data. Mean overlap values significantly lower than expected indicate that zonation is present and that different species tend to live at different elevations; overlap variances that are significantly higher than expected also indicate that zonation is present, but suggest that multiple species inhabit each zone. If the calculated overlap and variance values do not differ significantly from the corresponding null‐model values, then species distribution in the community is random (Ellison et al., 2000). All analyses were performed using EcoSim software 7.71 (Gotelli & Entsminger, 2005).

To determine whether elevation was correlated with species distributions, each floral and faunal species (mangrove plants, molluscs, and crabs) represented by ≥10 individuals was modeled separately. We tallied the abundance of each species along each transect separately, and then calculated the mean elevation for each species in different transects. The relationship between species distribution and surface elevation was quantified based on the average elevation of each species along each of the six transects. Significant variations in elevation among species were identified using one‐way analysis of variance (ANOVAs), followed by Duncan's post hoc tests. Data were log‐ or square‐root transformed when necessary to meet assumptions of normality and homogeneity of variances prior to statistical analyses.

As it was difficult to meet the assumptions of normality and homogeneity of variances for some of the data, we also used the non‐parametric Kruskal‐Wallis test, followed by stepwise step‐down comparisons to compare the densities of species at different elevations among sampling sites. Spearman's rank correlation coefficient was also used to examine the relationships between mangrove floral and faunal distributions, based on species abundances at each sampling site. These analyses were performed using SPSS v26.0 (IBM Corp.). Line charts and box plots were generated using Origin v9.5.1 (OriginLab Corp).

## 3 RESULTS

### 3.1 Floral and faunal assemblages

**FIGURE 4:**
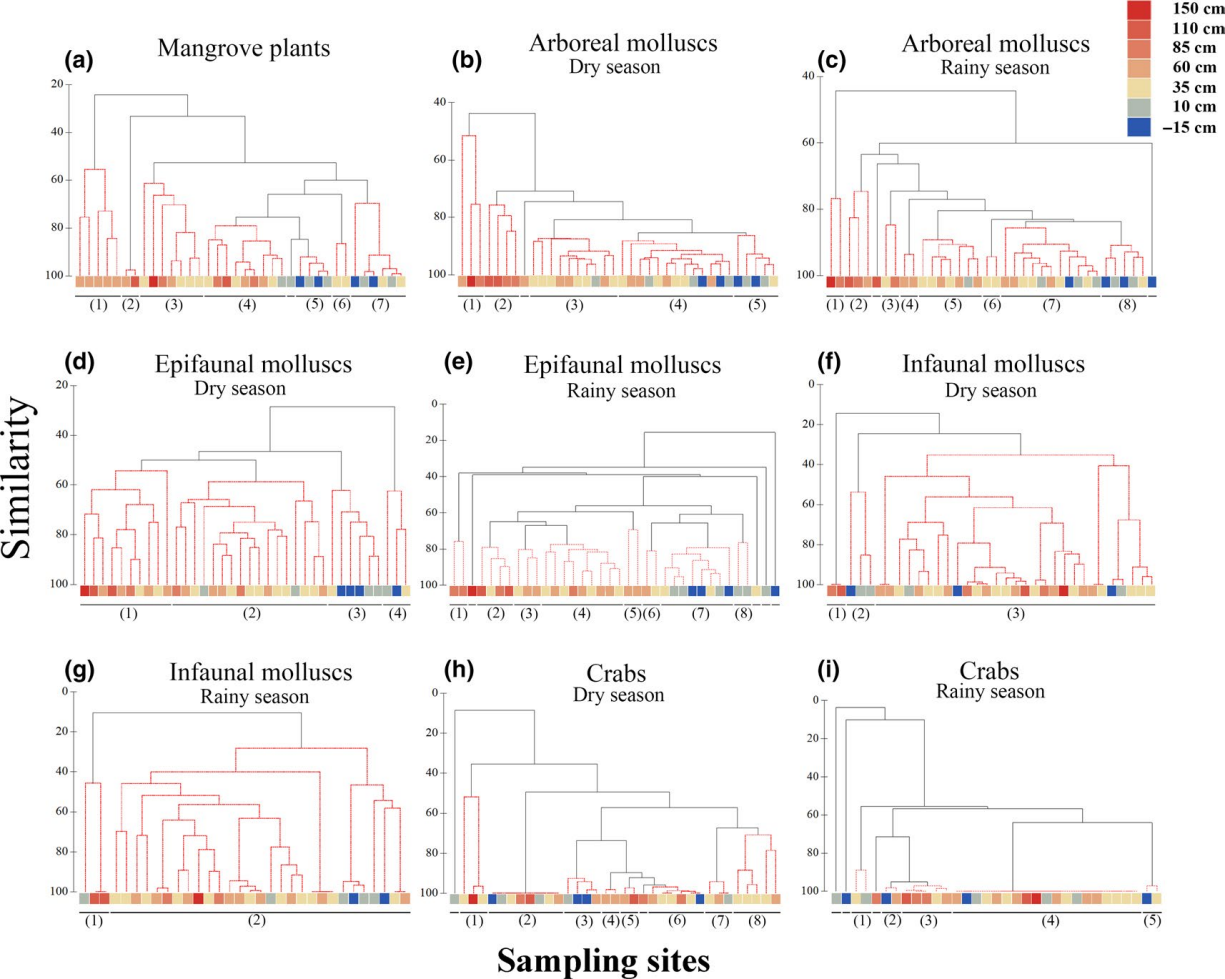
SIMPROF cluster analyses of (a) mangrove plants, (b) arboreal molluscs in the dry season, (c) arboreal molluscs in the wet season, (d) epifaunal molluscs in the dry season, (e) epifaunal molluscs in the wet season, (f) infaunal molluscs in the dry season, (g) infaunal molluscs in the wet season, (h) crabs in the dry season, and (i) crabs in the wet season. Significant clusters are shown as groups of red lines (*p* < .05). Leaves show the sampling sites and the elevation (−15–150 centimeters above mean sea level). Colors correspond to elevation

Cluster analyses indicated that mangrove plants were comprised of seven distinct assemblages (SIMPROF: *p* < .05) corresponding to the elevation gradient (Figure 4a). SIMPER analyses identified the species characterizing each observed assemblage. The first‐order species characterizing Group 1 was *Bruguiera gymnorhiza* (contribution rate: 84.55%), at an elevation of 60 cm, while the first‐order species characterizing Groups 2 and 3 was *Avicennia marina* (contribution rate: 57.40% and 41.10%, respectively), with an elevation range of 35–150 cm. The first‐order species characterizing Groups 4–7 was *Aegiceras corniculatum* (contribution rate: 59.07%, 78.39%, 35.59%, and 78.83%, respectively) at elevations of −15–110 cm.

The arboreal molluscs were also distributed in distinct faunal groups along the elevation gradient, irrespective of season (Figure 4b,c). In the dry season, the arboreal molluscs were clustered into five assemblages (SIMPROF: *p* < .05). The first‐order species characterizing Group 1 was *Cerithidea ornata* (contribution rate: 52.64%) at elevations of 60, 85, and 150 cm. The first‐order species characterizing Groups 2–4 was *Littoraria melanostoma* (contribution rate: 39.61%, 28.13%, and 31.61%, respectively) at elevations of −15–110 cm. The first‐order species characterizing Group 5 was *Littoraria scabra* (contribution rate: 34.94%) at elevations of −15–35 cm.

In the rainy season, the arboreal molluscs fell were classified into eight assemblages (SIMPROF: *p* < .05). The first‐order species characterizing Group 1 was *C*. *ornata* (contribution rate: 37.40%) at elevations of 85 cm and 150 cm. The first‐order species characterizing Groups 2, 3, 5, and 7 was *L*. *melanostoma* (contribution rate: 43.07%, 25.20%, 27.54%, and 29.27%, respectively) at elevations of −15–110 cm. The first‐order species characterizing Groups 6 and 8 was *L*. *scabra* (contribution rate: 26.85% and 41.26%, respectively) at elevations of −15–35 cm. The first‐order species characterizing Group 4 was *Enigmonia aenigmatica* (27.39%) at an elevation of 60 cm.

The epifaunal molluscs also formed well‐defined clusters (Figure 4d,e). In the dry season, epifaunal molluscs were clustered into four assemblages (SIMPROF: *p* < .05). The first‐order species characterizing Group 1 was *Cerithidea microptera* (contribution rate: 43.44%) at elevations of 35–150 cm. The first‐order species characterizing Group 2 was *Stenothyra japonica* (contribution rate: 20.09%) at elevations of 35–85 cm. The first‐order species characterizing Group 3 was *Cerithidea largillierti* (contribution rate: 30.59%) and the first‐order species characterizing Group 4 was *Cerithidea cingulata* (contribution rate: 58.71%), with both these assemblages found at elevations of −15–35 cm.

In the rainy season, epifaunal molluscs fell into eight assemblages (SIMPROF: *p* < .05). The first‐order species characterizing Group 1 was *Assiminea latericea* (contribution rate: 39.24%) at elevations of 35–85 cm. The first‐order species characterizing Groups 2, 4, and 7 was *C*. *microptera* (contribution rate: 43.34%, 23.37%, and 28.72%, respectively) at elevations of −15–110 cm. The first‐order species characterizing Group 3 was *S*. *japonica* (contribution rate: 22.67%) at elevations of 35–60 cm. The first‐order species characterizing Group 5 was *Neritina violacea* (contribution rate: 24.24%) at an elevation of 60 cm. *Cerithidea largillierti* was the first‐order species characterizing Groups 6 and 8 (contribution rate: 42.88% and 55.61%, respectively) at elevations of 10–60 cm.

Most of the infaunal molluscs fell into a single cluster (SIMPROF: *p* > .05) (Figure 4f,g). In the dry season, three clusters were identified (SIMPROF: *p* < .05). The only species characterizing Group 1 was *Geloina coaxans* (contribution rate: 100%) at elevations of 85–110 cm. The first‐order species characterizing Group 2 was *Pinguitellina cycladifomis* (contribution rate: 79.77%) at elevations of −15–10 cm. The first‐ and second‐order species characterizing Group 3 were *Indoaustriella scarlatoi* (contribution rate: 42.97%) and *Indoaustriella plicifera* (contribution rate: 42.88%), respectively, found at all elevations.

In the rainy season, only two clusters of infaunal molluscs were identified (SIMPROF: *p* < .05). The only species characterizing Group 1 was again *G*. *coaxans* (contribution rate: 100%), at elevations of 10–110 cm. The first‐ and second‐order species characterizing the second cluster were *I*. *plicifera* (contribution rate: 44.19%) and *I*. *scarlatoi* (contribution rate: 40.56%), respectively, and were found at all elevations.

Although our analyses of the crab fauna recovered eight clusters in the dry season and five clusters in the rainy season, the distribution of these clusters did not seem to be related to surface elevation (Figure 4h,i). In the dry season, the first‐order species characterizing Group 1 was *Sesarma plicata* (contribution rate: 76.75%) at elevations of 35 and 150 cm. The first‐order species characterizing Groups 2, 3, 5, 6, 7 and 8 was *Cleistostoma dilatatum* (contribution rate: 100.00%, 39.60%, 65.76%, 57.13%, 51.98%, and 44.81%, respectively) at elevations of −15–110 cm. *Paracleistostoma cristatum* was the first‐order species characterizing Group 4 (contribution rate: 50.92%) at an elevation of 60 cm.

In the rainy season, *C*. *dilatatum* was the first‐order species characterizing all the Groups (contribution rate: 56.19%, 56.09%, 61.15%, 100%, and 64.51%, respectively) at all elevations.

### 3.2 Zonation patterns

Randomization tests indicated that the mangrove plants exhibited significant zonation (Table 1). The mean overlap was low and did not differ significantly from the null model (*p* = .39), but the variance in overlap was significantly higher that would be expected by random chance (*p* = .02). The mean overlaps and variance in overlaps were significantly higher (*p* < .05) for all mollusc groups (i.e., arboreal, epifaunal, and infaunal) than randomizations regardless of season (Table 1). These results suggested that mangrove molluscs exhibited significant zonation, but with more overlap than expected. Randomization tests also indicated that mangrove crabs were not significantly zoned in the dry season (Table 1), as neither the mean overlap nor the variance were significantly different from random (*p* > .05). In the rainy season, the mean overlap of the mangrove crabs was significantly higher than predicted by randomizations (*p* = .009); the variance in overlap was not tested in the rainy season because only two species of crabs were collected.

**TABLE 1:** Pianka's indices of overlap based on species abundance at each sampling site, with niche breadth retained and reshuffled zero states

Species	Season	Mean overlap		Variance	
		Observed	Simulated	Observed	Simulated
Mangrove plants		0.180	0.191	0.066*	0.034
Arboreal molluscs	D	0.324*	0.270	0.103***	0.031
	R	0.311*	0.246	0.105***	0.033
Epifaunal molluscs	D	0.192*	0.155	0.050***	0.026
	R	0.178*	0.121	0.073**	0.028
Infaunal molluscs	D	0.427*	0.131	0.095*	0.024
	R	0.224*	0.136	0.077*	0.025
Crabs	D	0.340	0.298	0.046	0.027
	R	0.519*	0.271	0.000	0.000

Note

The mean and variance of Pianka's index of overlap were both compared with a null model generated using Randomization Algorithm 3 (RA3), based on 1,000 random samplings of the abundance data (Ellison et al., 2000). Asterisks correspond to significance: *α = 0.05; **α = 0.01; ***α = 0.001.

Abbreviations: D, dry season; R, rainy season.

### 3.3 Species distributions

**FIGURE 5:**
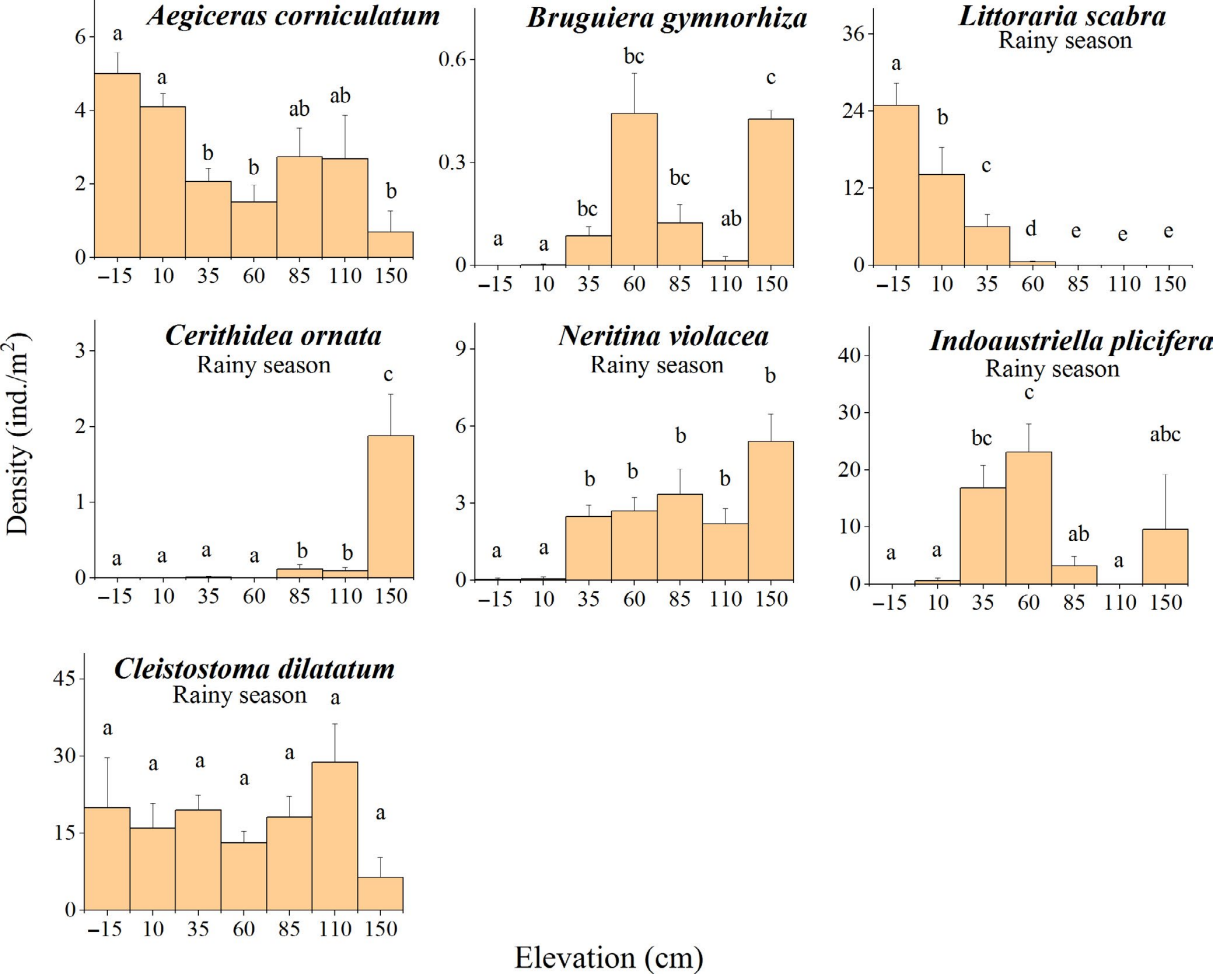
Mean densities (±*SE*) of seven typical species at different elevations based on density data from each sampling site. Bars labeled with different lowercase letters are significantly different (*p* < .05)

**FIGURE 6:**
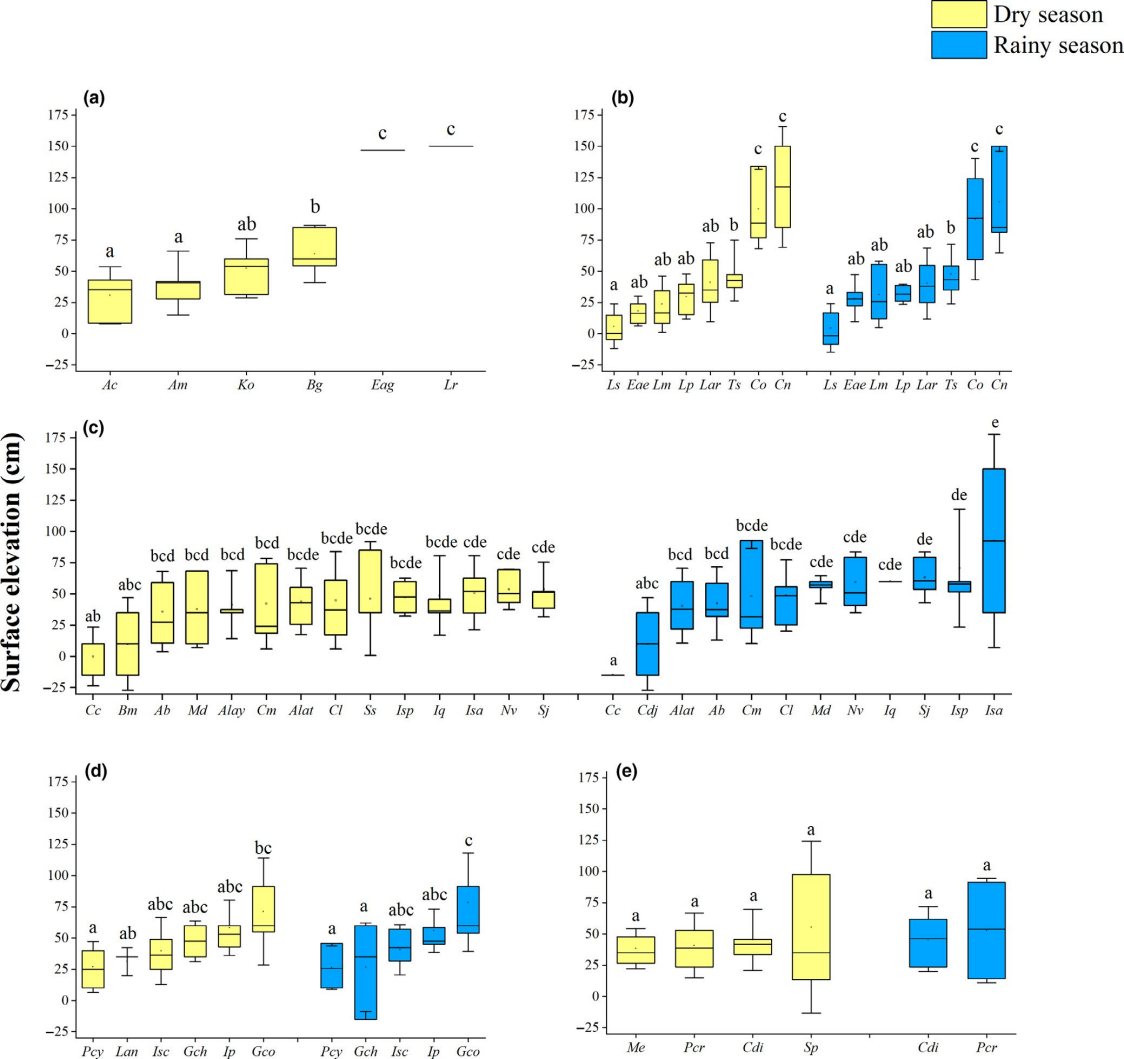
Boxplots showing the mean surface elevations (in centimeters above mean sea level) of (a) the most abundant mangrove plants, (b) arboreal molluscs, (c) epibenthic molluscs, (d) infaunal molluscs, and (e) infaunal crabs. Yellow boxes represent the dry season, while blue boxes represent the rainy season. The midline of each box indicates the mean; the open square indicates the median. The means of boxes labeled with different lowercase letters are significantly different (*p* < .05). Species with fewer than 10 samples were omitted. Species abbreviations: *Ac*, *Aegiceras corniculatum*; *Am*, *Avicennia marina*; *Ko*, *Kandelia obovata*; *Bg*, *Bruguiera gymnorhiza*; *Eag*, *Excoecaria agallocha*; *Lr*, *Lumnitzera racemosa*; *Ls*, *Littoraria scabra*; *Eae*, *Enigmonia aenigmatica*; *Lm*, *Littoraria melanostoma*; *Lp*, *Littoraria pallescens*; *Lar*, *Littoraria ardouiniana*; *Ts*, *Terebralia sulcata*; *Co*, *Cerithidea ornata*; *Cn*, *Cassidula nucleus*; *Cc*, *Cerithidea cingulata*; *Bm*, *Batillaria multiformis*; *Ab*, *Assiminea brevicula*; *Md*, *Mainwaringia dantaae*; *Alay*, *Allochroa layardi*; *Cm*, *Cerithidea microptera*; *Alat*, *Assiminea latericea*; *Cl*, *Cerithidea largillierti*; *Ss*, *Salinator sanchezi*; *Isp*, *Iravadia* sp.; *Iq*, *Iravadia quadrasi*; *Isa*, *Iracadia sakaguchii*; *Nv*, *Neritina violacea*; *Sj*, *Stenothyra japonica*; *Cdj*, *Cerithidea djadjariensis*; *Pcy*, *Pinguitellina cycladifomis*; *Lan*, *Laternula anatina*; *Isc*, *Indoaustriella scarlatoi*; *Gch*, *Glauconome chinensis*; *Ip*, *Indoaustriella plicifera*; *Gco*, *Geloina coaxans*; *Me*, *Macrophthalmus erato*; *Pcr*, *Paracleistostoma cristatum*; *Cdi*, *Cleistostoma dilatatum*; *Sp*, *Sesarma plicata*

Across all sites, we counted 8,149 mangrove trees (>0.5 m) in seven species. The four most abundant species were *A*. *corniculatum* (*n* = 6,927), *Kandelia obovata* (*n* = 404), *Bruguiera gymnorhiza* (*n* = 388), and *A*. *marina* (*n* = 384). Kruskal‐Wallis tests showed that the densities of most mangrove plants differed significantly among elevations (*p* < .05), with the exception of *K*. *obovata* (*H* = 8.901, *df* = 6, *p* > .05). In addition, the elevation corresponding to maximum density differed among species (Appendix S1). For example, *A*. *corniculatum* was significantly denser at elevations of −15 cm and 10 cm than at 35, 60, and 150 cm (*H* = 27.504, *df* = 6, *p* < .05; Figure 5a), while *Excoecaria agallocha* was significantly denser at 150 cm than at any other elevation (*H* = 31.684, *df* = 6, *p* < .001; Figure 5b). One‐way ANOVAs indicated that mean elevation differed significantly among mangrove plant species (Figure 6a) (*F* = 15.801; *df* = 5; *p* < .05). For example, *A*. *corniculatum* was found in the lower‐mid‐tidal zone, with an average elevation of 24.84 ± 18.28 cm, while *A*. *marina* and *K*. *obovata* were most common in the mid‐tidal zone, with mean elevations of 31.03 ± 13.22 and 45.07 ± 15.46 cm, respectively. *Bruguieragymnorhiza* grew at a mean elevation of 59.68 ± 21.45 cm, significantly higher than *A*. *corniculatum* and *A*. *marina* (ANOVA, Duncan's post‐hoc test, *p* < .05). We identified two additional mangrove plants in the upper intertidal, *E*. *agallocha* (*n* = 20) and *Lumnitzera racemosa* (*n* = 23), with mean elevations of 146.75 cm and 150.00 cm, respectively. These plants were found at significantly higher elevations than other mangrove plants (ANOVA, Duncan's post‐hoc test, *p* < .001). A third species, *Acanthus ilicifolius*, was also found at 150 cm, but this species was omitted from this study due to small number of individuals found (*n* < 10).

The distributions of arboreal molluscs also showed obvious patterns of zonation correlating with surface elevation (Appendix S1; Figure 5b). The Littorinidae were the dominant family of arboreal molluscs, followed by the Potamididae and the Ellobiidae. Kruskal–Wallis tests showed that the densities of 87.5% of the arboreal molluscs differed significantly among elevations (*p* < .05; Appendix S1). For example, in the rainy season, the density of *L*. *scabra* was significantly greater at −15 cm than at all other elevations (*H* = 71.877, *df* = 6, *p* < .0001), with the density decreasing as elevation increased (Figure 5c). In contrast, *C*. *ornata* was significantly denser at 150 cm than at all other elevations in the rainy season (*H* = 66.002, *df* = 6, *p* < .0001); with density decreasing with elevation (Figure 5d). One‐way ANOVAs identified significant differences in mean elevation among arboreal molluscs (*F* = 7.794; *df* = 15; *p* < .001) (Figure 6b). In both the dry and rainy seasons, *L*. *scabra* was mainly distributed in the lower intertidal, significantly lower than *Terebralia sulcata* (ANOVA, Duncan's post‐hoc test, *p* < .05) and *C*. *ornata* (ANOVA, Duncan's post‐hoc test, *p* < .001). *Terebralia sulcata* was found significantly lower than *C*. *ornata* (ANOVA, Duncan's post‐hoc test, *p* < .05) and *Cassidula nucleus* (ANOVA, Duncan's post‐hoc test, *p* < .01), both of which were found close to the mean high‐water line. There were no significant differences in the surface elevations of arboreal molluscs between the dry and rainy seasons (ANOVA, Duncan's post‐hoc test, *p* > .05).

Epifaunal mollusc distributions were also associated with elevation (Appendix S1; Figure 6c). Kruskal–Wallis tests showed that the densities of 69.9% of the epifaunal molluscs differed significantly among elevations (*p* < .05; Appendix S1). For example, in the rainy season, densities of *N*. *violacea* were significantly lower at −15 and 10 cm than at all other elevations (*H* = 63.872, *df* = 6, *p* < .0001; Figure 5e). One‐way ANOVAs identified significant differences in mean elevation among epifaunal molluscs (*F* = 1.686; *df* = 25; *p* < .05; Figure 6c). In particular, the genus *Cerithidea* was widely distributed in the rainy season, with *C*. *cingulata* found close to the MSL, significantly lower than *C*. *largillierti* and *C*. *microptera* (ANOVA, Duncan's post‐hoc test, *p* < .05). *Iracadia sakaguchii*, *N*. *violacea*, and *S*. *japonica* were found at the highest elevations in both seasons, significantly higher than *C*. *cingulata* (ANOVA, Duncan's post‐hoc test, *p* < .05). There were no significant differences in the surface elevations of epifaunal molluscs between the dry and rainy seasons (ANOVA, Duncan's post‐hoc test, *p* > .05).

Few species of infaunal molluscs and crabs were collected in this study (Figure 5d,e). Kruskal–Wallis tests showed that the densities of 72.7% of the infaunal molluscs differed significantly among elevations (*p* < .05; Appendix S1). For example, in the rainy season, *I*. *plicifera* was densest at an elevation of 60 cm, being significantly denser than at −15, 10, 85, and 110 cm (*H* = 49.091, *df* = 6, *p* < .0001; Figure 5f). One‐way ANOVAs identified significant differences in mean elevation among infaunal molluscs (*F* = 2.204; *df* = 10; *p* < .05; Figure 6d). *Pinguitellinacycladifomis* was found at significantly lower elevations than *G*. *coaxans* in both seasons (ANOVA, Duncan's post‐hoc test, *p* < .05), while *Glauconome chinensis* was significantly lower than *G*. *coaxans* in the rainy season only (ANOVA, Duncan's post‐hoc test, *p* < .05). There were no significant differences in the surface elevations of infaunal molluscs between the dry and rainy seasons (ANOVA, Duncan's post‐hoc test, *p* > .05).

Kruskal–Wallis tests showed that the densities of 66.7% of the infaunal crabs did not differ significantly among elevations (*p* > .05; Appendix S1). For example, in the rainy season, *C*. *dilatatum* was widely distributed, with no significant differences in density among elevations (*H* = 8.418, *df* = 6, *p* = .209; Figure 5g). One‐way ANOVAs identified no significant differences in the mean elevations of infaunal crabs in either season (*F* = 0.168; *df* = 5; *p* > .05). In addition, there were no significant differences in the surface elevations of infaunal crabs between the dry and rainy seasons (ANOVA, Duncan's post‐hoc test, *p* > .05).

### 3.4 Co‐occurrence

Several significant correlations were identified among mangrove species (Table 2). A significant negative correlation was found between *A*. *corniculatum* abundance and *B*. *gymnorhiza* abundance (*ρ* = −0.55, *p* < .001), while a significant positive correlation was found between *E*. *agallocha* abundance and *L*. *racemose* abundance (*ρ* = 0.72, *p* < .001). The abundances of some molluscs were also significantly correlated with the abundances of certain mangrove plants (Table. 2). For example, the abundance of *L*. *melanostoma* was significantly positively correlated with that of *A*. *corniculatum* (*ρ* = 0.49, *p* = .003 in the rainy season; *ρ* = 0.50, *p* = .002 in the dry season), while the abundance of *L*. *melanostoma* was significantly negatively correlated with that of *B*. *gymnorhiza* (*ρ* = −0.38, *p* = .024 in the rainy season; *ρ* = −0.42, *p* = .011 in the dry season). The abundance of *C*. *ornata* was significantly positively correlated with that of *E*. *agallocha* (*ρ* = 0.47, *p* = .004 in the rainy season; *ρ* = 0.51, *p* = .001 in the dry season). The abundances of *N*. *violacea* and some other epifaunal molluscs were significantly positively correlated with the abundance of *A*. *marina* (*ρ* = 0.37, *p* = .028 for *N*. *violacea* in the rainy season; *ρ* = 0.36, *p* = .032 for *N*. *violacea* in the dry season; Table 2). The abundance of *I*. *plicifera* was also significantly positively correlated with that of *A*. *marina* (*ρ* = 0.44, *p* = .008 in the rainy season; *ρ* = 0.54, *p* = .001 in the dry season). The only significant relationships involving infaunal crabs were the positive correlations between *C*. *dilatatum* abundance and *K*. *obovata* abundance in the rainy season (*ρ* = 0.36 *p* = .029), and between *C*. *dilatatum* abundance and *A*. *marina* abundance in the dry season (*ρ* = 0.37 *p* = .025).

**TABLE 2:** Spearman's rank correlation coefficients between pairs of mangrove plants, and between pairs of mangrove plants and molluscs or crabs; correlations were calculated based on species abundance data from each sampling site

Species		Season	*Ac*	*Am*	*Ko*	*Bg*	*Eag*	*Lr*
Mangrove plants	*Ac*		–	−0.06	0.05	−0.55***	−0.06	−0.11
	*Bg*		−0.55***	−0.25	0.07	–	0.22	0.22
	*Eag*		−0.06	−0.23	−0.11	0.22	–	0.72***
	*Lr*		−0.11	−0.16	−0.19	0.22	0.72***	–
Arboreal molluscs	*Lm*	D	0.50**	−0.04	0.05	−0.42*	−0.2	−0.28
		R	0.49**	0.00	0.06	−0.38*	−0.03	−0.28
	*Lp*	D	0.28	0.35*	−0.18	−0.24	−0.16	−0.07
		R	0.11	0.29	0.03	−0.36*	−0.29	−0.24
	*Ls*	D	0.24	0.18	−0.21	−0.46**	−0.33*	−0.26
		R	0.26	0.13	−0.25	−0.50**	−0.34*	−0.26
	*Co*	D	0.05	0.12	0.01	0.10	0.51**	0.39
		R	0.02	0.18	−0.1	0.07	0.47*	0.39*
	*Cn*	D	0.05	−0.04	−0.04	0.04	0.49**	0.7***
		R	−0.08	0.21	−0.12	0.21	0.31	0.48*
	*Eae*	D	0.18	0.2	−0.35*	−0.16	−0.34*	−0.26
Epifaunal molluscs	*Cc*	D	−0.07	0.21	−0.34	−0.29	−0.07	−0.05
	*Cl*	R	−0.11	−0.06	0.38	0.25	−0.12	−0.07
	*Ab*	D	0.39*	0.14	0.12	0.06	−0.24	−0.17
		R	−0.03	−0.32	0.23	0.37*	−0.28	−0.19
	*Iq*	R	−0.47**	−0.34*	−0.11	0.50**	−0.09	−0.06
	*Nv*	D	−0.3	0.36*	−0.15	0.35*	0.07	0.11
		R	−0.27	0.37*	−0.13	0.35*	0.22	0.23
	*Sj*	D	−0.26	0.45**	0.00	0.31	−0.19	−0.21
		R	−0.29	0.38*	−0.05	0.26	0.13	0.16
	*Md*	D	−0.3	0.49**	−0.31	0.03	−0.11	−0.08
		R	−0.05	0.45**	0.03	−0.06	−0.13	−0.09
	*Isa*	D	−0.03	0.44**	0.03	0.14	−0.09	−0.16
		R	−0.16	0.07	−0.15	0.16	0.47**	0.68***
	*Isp*	D	−0.21	0.35*	−0.21	0.27	−0.09	−0.06
		R	−0.14	0.26	−0.06	0.18	0.21	0.37*
Infaunal molluscs	*Ip*	D	−0.24	0.54***	−0.41*	0.10	−0.08	0.08
		R	−0.23	0.44**	−0.18	0.29	−0.07	0.08
	*Gco*	D	−0.36*	0.07	0.11	0.50**	0.3	0.31
		R	−0.18	0.28	−0.14	0.17	0.18	0.35*
	*Pcr*	D	−0.06	0.10	−0.35*	−0.26	−0.14	−0.1
Crabs	*Cdi*	D	0.07	0.37*	0.24	0.00	−0.25	−0.21
		R	0.04	0.10	0.36*	−0.13	−0.03	−0.15

Note

Asterisks correspond to significance: *α = 0.05; **α = 0.01; ***α = 0.001.

Species abbreviations: *Ac*, *Aegiceras corniculatum*; *Am*, *Avicennia marina*; *Ko*, *Kandelia obovata*; *Bg*, *Bruguiera gymnorhiza*; *Eag*, *Excoecaria agallocha*; *Lr*, *Lumnitzera racemosa*; *Ls*, *Littoraria scabra*; *Eae*, *Enigmonia aenigmatica*; *Lm*, *Littoraria melanostoma*; *Lp*, *Littoraria pallescens*; *Co*, *Cerithidea ornata*; *Cn*, *Cassidula nucleus*; *Cc*, *Cerithidea cingulata*; *Ab*, *Assiminea brevicula*; *Md*, *Mainwaringia dantaae*; *Cl*, *Cerithidea largillierti*; *Isp*, *Iravadia* sp.; *Iq*, *Iravadia quadrasi*; *Isa*, *Iracadia sakaguchii*; *Nv*, *Neritina violacea*; *Sj*, *Stenothyra japonica*; *Pcy*, *Pinguitellina cycladifomis*; *Ip*, *Indoaustriella plicifera*; *Gco*, *Geloina coaxans*; *Cdi*, *Cleistostoma dilatatum*.

Abbreviations: D, dry season; R, rainy season.

## 4 DISCUSSION

### 4.1 Relationships between species distributions and surface elevation

Our results indicated that mangrove plants in Zhenzhu Bay exhibited distinct zonation patterns, where different species tended to inhabit different areas along the elevation gradient. The mangrove *Aegiceras corniculatum* had the lowest mean elevation, followed by *Avicennia marina*, *Kandelia obovata*, *Bruguiera gymnorhiza*, *Excoecaria agallocha* and *Lumnitzera racemosa*. This was consistent with the surface‐elevation‐correlated zonation patterns observed in other natural mangrove forests (Fu et al., 2019; Wang, Li, & Wang, 2019; Zhu et al., 2019).

Previous studies have shown that hydroperiods may affect mangrove plant distributions (Crase et al., 2013; He et al., 2007; Leong et al., 2018; Watson, 1928), as mangrove plants have species‐specific hydroperiod tolerance thresholds that are determined by surface elevation (Ball, 1988a). *Aegiceras corniculatum* grew in the lower intertidal zone, at the seaward edge of the forest. This was not surprising, as *A*. *corniculatum* is known as a pioneer mangrove species (Cheng, Wang, Fei, Jiang, & Ye, 2015) and is well adapted to the lower intertidal due to growth strategies like stem elongation (He et al., 2007). In contrast, *E*. *agallocha* was distributed above the MHW, possibly because the buoyancy of *E*. *agallocha* seeds allowed this species to reach the landward edge of the forest (Ye, Lu, Wong, & Tam, 2004). Additionally, the mean elevation of *A*. *corniculatum* was significantly lower than that of *B*. *gymnorhiza* (ANOVA, Duncan's post‐hoc test, *p* < .05), and the abundances of these two species were significantly negatively correlated (*ρ* = −0.55, *p* < .001). This might have been because *B*. *gymnorhiza* is more tolerant of low light than *A*. *corniculatum* (Peng et al., 2016), and increasing densities of *B*. *gymnorhiza* communities at higher elevations limited the available light irradiance, and thus the growth of *A*. *corniculatum*.

Salinity may also strongly affect the distributions of mangrove plants (Barik et al., 2018; Crase et al., 2013; Xu, Wang, Xin, Liu, & Wang, 2020) and may be particularly high at the landward edge of the mangrove forest, as evaporation concentrates the salts at locations of higher elevation (Smith, 1992). Indeed, we found a significant positive correlation between soil salinity and surface elevation (*ρ* = 0.36, *p* < .001). In addition, soil salinity is also determined by distance to local freshwater inputs from runoff (Duke, Ball, & Ellison, 1998). Due to the steady inflow of fresh water, salinity levels at the landward edge of the forest were not extreme. Soil salinities did not differ significantly along the elevation gradient between 35 cm and 150 cm (*H* = 4.916, *df* = 6, *p* = .296; Appendix S2). Thus, salinity had little effect on the distributions of mangrove plants. As a mangrove plant with strong salt tolerance (Ball, 1988b; Burchett, Clarke, Field, & Pulkownik, 1989), only the abundance of *A*. *marina* was significantly positively correlated with soil salinity (*ρ* = 0.347, *p* = .038; Appendix S3).

Similar to the mangrove plants, distributions of arboreal, epifaunal, and infaunal molluscs in the mangrove forest showed obvious patterns of zonation correlated with surface elevation. To the best of our knowledge, this is the first study to explicitly quantify the influences of surface elevation on the spatial distributions of mangrove fauna within the intertidal zone.

This distinct mollusc zonation patterns along the elevation gradient in Zhenzhu Bay might be caused by the sensitivity of different molluscs to environmental factors associated with tidal inundation. The Littorinidae, Ellobiidae, and Potamididae are three of the very few molluscan families that have high fidelity to mangrove forests (Cantera, Arnaud, & Thomassin, 1983; Reid, Dyal, Lozouet, Glaubrecht, & Williams, 2008; Reid, Dyal, & Williams, 2010). Species in these families are common in mangrove forests and often show zonation patterns that partially parallel those of mangrove plants (Ellison, Farnsworth, & Merkt, 1999). In Zhenzhu Bay, *Littoraria* species, except for *Littoraria scabra*, were widely distributed vertically. *Littoraria scabra*, which is a typical oceanic species (Reid, 1985), was restricted to the lower intertidal zone. Previous studies have also primarily observed *L*. *scabra* at the seaward edges of mangrove forests (Torres et al., 2008), possibly because this species is intolerant of turbid water or sediments on the substrate (Reid, 1985).

In contrast to *L*. *scabra*, *Cassidula nucleus* was found in the upper intertidal, significantly higher than all other molluscs except *Cerithidea ornata*. This distribution might be driven by the availability of food in the upper intertidal (Peng, Zhang, & Lee, 2017). Importantly, the lengthy periods of inundation characteristic of the lower intertidal zone are fatal to halophile ellobiids, which lack an operculum and have lungs adapted to air‐breathing (Martins, 2001; Morton & Graham, 1955; Ragionieri et al., 2015). Mollusc distributions are also affected by salinity (Montagna, Estevez, Palmer, & Flannery, 2008). For example, the gastropod genus *Iravadia*, which inhabits brackish water, was found in some areas of the higher intertidal, at the landward edge of the forest. In this area, the surface water was diluted due to the steady freshwater inflow from the supratidal zone (Brown & Gallagher, 1985; Kobayashi & Wada, 2004).

Although a clear pattern of zonation was observed across the mangrove molluscs, there were still some overlaps in the elevations of some species, which may have been caused by different driving factors. *Cerithidea ornata* was found at same elevation as *C*. *nucleus* in Zhenzhu Bay, with a high niche overlap. Tree‐climbing behaviors are common in *Cerithidea* species, especially *C*. *ornata*. These snails anchor themselves to mangrove trunks at various heights using mucus during high tides, in order to escape predation and avoid physiological stress (McGuinness, 1994; Reid, 2014; Reid et al., 2008; Vannini, Rorandelli, Lähteenoja, Mrabu, & Fratini, 2006). For infaunal molluscs, food availability may be the major factor driving vertical distributions. Similar to *L*. *scabra*, *Pinguitellina cycladifomis* was mainly distributed along the seaward margin of the mangrove forest. This detritus feeder consumes decomposing bacteria and plant debris brought in by the tides (Xu & Zhang, 2011) and was found at the same elevation as *L*. *scabra* and other molluscs restricted to the lower intertidal.

Additionally, some molluscan distributions were likely affected by tree species abundance. For example, the abundance of *Littoraria melanostoma* was positively correlated with the abundance of *A*. *corniculatum*, and negatively correlated with the abundance of *B*. *gymnorhiza*. Most littorinids occur on tree leaves, as opposed to trunks or branches, and are found at heights ranging from near‐ground to >2 m (Lee & Williams, 2002). *Littoraria melanostoma* is found lower on trees than other littorinids, such as *Littoraria*. *ardouiniana* (Lee & Williams, 2002). In Zhenzhu Bay, *A*. *corniculatum* is a low‐growing tree (Wang & Wang, 2007) and tends to attain heights of ~2 m with a low crown base height (Liu, Fan, & Li, 2012). Thus, this tree provides a suitable habitat for *L*. *melanostoma*. Conversely, *B*. *gymnorhiza* is a taller tree of ~3 m with a high crown base height in Zhenzhu Bay (Liu et al., 2012). Thus, the leaves of this tree are >2 m in height, making this tree unsuitable for most mangrove littorinids. In addition, the abundances of some epifaunal molluscs were significantly positively correlated with the abundance of *A*. *marina*. The three‐dimensional settlement structures provided by *A*. *marina* pneumatophores create sandier sediments that promote relatively high faunal density and diversity. In addition, *A*. *marina* pneumatophores tend to trap high volumes of drift algae, which acts as food for some epifaunal molluscs (Alfaro, 2006).

In contrast to molluscs, crabs in the mangrove forest were not separated into clear regions associated with surface elevation, and no visible zonation patterns in species distributions were observed along the elevation gradient. Previous studies have shown that mangrove crabs also exhibit zonation patterns, and that these patterns are related to multiple factors, including salinity fluctuations, degree of tidal inundation, and feeding modes (Dahdouh‐Guebas et al., 2002; Machiwa & Hallberg, 1995; Ragionieri et al., 2015). However, crabs are extremely active and it is impossible to collect swimming or tree‐dwelling crabs using the traditional quadrat method employed in this study. In addition, many mangrove crabs adopt a nocturnal lifestyle to escape high temperatures and/or predation (Micheli, Gherardi, & Vannini, 1991). Because we collected few individual crabs belonging to only a few species, our sampling technique may not accurately reflect crab abundance and diversity. More representative samples are required to further investigate zonation patterns of crab populations in the mangrove forests of Zhenzhu Bay.

### 4.2 Seasonal changes in zonation patterns

Our cluster analyses indicated limited difference among assemblages of all groups of organisms between seasons. Only the number of assemblages varied, but the primary characterizing species, and the distributions of species among elevations, were generally similar between the dry and rainy seasons. In addition, there were no significant differences in the surface elevations of mollusc or crab species between the dry and rainy seasons. Thus, there were no obvious seasonal changes in the vertical zonation patterns of the fauna in Zhenzhu Bay. Zhenzhu Bay is located in a subtropical region, with seasonal differences in temperature and precipitation (EBCBS, 1993). Although such seasonal variations typically affect the reproductive activity and species density of mangrove fauna (Beasley et al., 2005; Ng & Williams, 2012), mangrove molluscs usually have long life spans (Black & Johnson, 2001) and food searching strategies and migratory tidal behaviors may be maintained by long‐lasting spatial inertia (Vannini, Cannicci, Mrabu, Rorandelli, & Fratini, 2008; Vannini, Coffa, Lori, & Fratini, 2008). Similarly, adult intertidal crabs generally have restricted home ranges, and do not tend to wander about in their habitats (Vermeiren & Sheaves, 2015; Yamaguchi & Tabata, 2004). Thus, the relationships among flora, fauna and elevation were stable in the mangrove forests of Zhenzhu Bay between seasons.

### 4.3 Potential threats to mangrove forests due to rising sea levels

The most recent projections of the Intergovernmental Panel on Climate Change (Fifth Assessment Report; IPCC AR5) suggest a SLR of 0.28–0.98 m by 2,100 (Church et al., 2013). The increasing inundation associated with SLR threatens the stability of mangrove forests (Lovelock et al., 2015). In Zhenzhu Bay, almost all mangrove forests were distributed within the intertidal zone, at relative elevations between the MSL and the MHW (119 cm) (Figure 3). Because the tidal flat rises in the middle of the mangrove forest (Figure 3, Fan & Li, 1997), rising sea levels may isolate portions of the seaward edge of the existing mangrove forest, causing habitat fragmentation and decreasing biodiversity (Lee & Williams, 2002).

In addition, and about 85 percent of the mangrove forests in Zhenzhu Bay are seaward of artificial seawalls (Fan & Li, 1997) and therefore unable to escape SLR as landward migration is blocked by seawalls (Borchert, Osland, Enwright, & Griffith, 2018; Lovelock et al., 2015; McKee & Vervaeke, 2018). At the landward edge, SLR may thus reduce the availability of suitable elevations for mangrove flora and fauna, and lead to the disappearance of species restricted to the upper intertidal, including *E*. *agallocha*, *L*. *racemosa*, *C*. *ornata*, and *C*. *nucleus* (Fu et al., 2019). However, mangrove forest species distributions depend not only on local SLR, but also on the rate of surface elevation change (Ellison, 2008; Gilman, Ellison, Duke, & Field, 2008). To predict possible changes in species distributions in future studies, it is important to consider the relationship between rates of surface elevation change and SLR (Webb et al., 2013).

## 5 CONCLUSION

Almost all of the mangrove forests in Zhenzhu Bay were distributed within the intertidal zone, between the local MSL and the MHW. The spatial distributions of mangrove plants and mangrove‐associated molluscs in Zhenzhu Bay exhibited obvious patterns of zonation. Each of the arboreal, epifaunal, and infaunal molluscan species studied occupied distinct positions along the elevation gradient, irrespective of season. However, no correlations were found between crab distributions and surface elevations. To the best of our knowledge, this is the first study to explicitly quantify the influences of surface elevation on the spatial distributions of mangrove fauna within the intertidal zone. This characterization of the vertical ranges of various flora and fauna in mangrove forests provides a framework for future studies aimed at predicting changes in the structures and functions of mangrove forests in response to SLR. Our results will also help to guide mangrove conservation efforts.

## CONFLICT OF INTEREST

The authors declare no competing interests.

## AUTHOR CONTRIBUTIONS

**Wei Ma:** Conceptualization (lead); data curation (equal); formal analysis (equal); investigation (lead); methodology (lead); project administration (lead); software (equal); visualization (equal); writing–original draft (equal). **Wenqing Wang:** Conceptualization (lead); funding acquisition (lead); investigation (supporting); methodology (lead); project administration (lead); resources (lead); supervision (lead); validation (lead); writing–review and editing (lead). **Chaoyi Tang:** Conceptualization (supporting); investigation (supporting); methodology (supporting). **Guogui Chen:** Conceptualization (supporting); investigation (supporting); methodology (supporting). **Mao Wang:** Conceptualization (lead); funding acquisition (lead); methodology (lead); project administration (lead); resources (lead); supervision (lead); validation (lead); writing–review and editing (lead).

## Supporting information

Supplementary Material

## Data Availability

The data used in this study have been archived through Dryad online data repository and are publically available at https://doi:10.5061/dryad.s1rn8pk45.
