# Supplementary material for: Zonation of mangrove flora and fauna in a subtropical estuarine wetland based on surface elevation
Source: Ecol Evol. 2020 Jun 4;10(14):7404–18. doi: 10.1002/ece3.6467 (PMC7391335; doi:10.1002/ece3.6467)
Supplement: Supplementary file 1 — Supplementary Material [file ECE3-10-7404-s001.docx]

S1 Mean species density (individuals/m^2^) at different elevations, based on species densities at each sampling site. Means within a row labeled with different lowercase letters are significantly different (P < 0.05). Species abbreviations: *Ac, Aegiceras corniculatum; Am, Avicennia marina; Ko, Kandelia obovata; Bg, Bruguiera gymnorhiza; Eag, Excoecaria agallocha; Lr, Lumnitzera racemosa; Ls, Littoraria scabra; Eae, Enigmonia aenigmatica; Lm, Littoraria melanostoma; Lp, Littoraria pallescens; Lar, Littoraria ardouiniana; Ts, Terebralia sulcata; Co, Cerithidea ornata; Cn, Cassidula nucleus; Cc, Cerithidea cingulata; Bm, Batillaria multiformis; Ab, Assiminea brevicula; Md, Mainwaringia dantaae; Alay, Allochroa layardi; Cm, Cerithidea microptera; Alat, Assiminea latericea; Cl, Cerithidea largillierti; Ss, Salinator sanchezi; Isp, Iravadia* sp.*; Iq, Iravadia quadrasi; Isa, Iracadia sakaguchii; Nv, Neritina violacea; Sj, Stenothyra japonica; Cdj, Cerithidea djadjariensis; Pcy, Pinguitellina cycladifomis; Lan, Laternula anatina; Isc, Indoaustriella scarlatoi; Gch, Glauconome chinensis; Ip, Indoaustriella plicifera; Gco, Geloina coaxans; Me, Macrophthalmus erato; Pcr, Paracleistostoma cristatum; Cdi, Cleistostoma dilatatum; Sp, Sesarma plicata.* D, dry season; R, rainy season.

| Species | Season | Elevation (cm) | | | | | | |
| --- | --- | --- | --- | --- | --- | --- | --- | --- |
|  |  | -15 | 10 | 35 | 60 | 85 | 110 | 150 |
| *Ac* |  | 5.00a | 4.10a | 2.07b | 1.50b | 2.74ab | 2.68ab | 0.69b |
| *Am* |  | 0.05 | 0.10 | 0.18 | 0.23 | 0.11 | 0.13 | 0.00 |
| *Ko* |  | 0.01a | 0.04a | 0.24b | 0.13ab | 0.14ab | 0.29ab | 0.00a |
| *Bg* |  | 0.00a | 0.00a | 0.09bc | 0.44bc | 0.12bc | 0.01ab | 0.43c |
| *Eag* |  | 0.00a | 0.00a | 0.00a | 0.00a | 0.00a | 0.00a | 0.25b |
| *Lr* |  | 0.00a | 0.00a | 0.00a | 0.00a | 0.00a | 0.00a | 0.31b |
| *Ls* | D | 11.93a | 11.09a | 4.80b | 0.56c | 0.05d | 0.03d | 0.00d |
|  | R | 0.34a | 0.53a | 0.40a | 0.14b | 0.02bc | 0.00c | 0.00c |
| *Eae* | D | 13.65a | 11.09a | 7.82ab | 4.9bc | 3.02bc | 3.94bc | 0.08c |
|  | R | 0.28ab | 0.38a | 0.45a | 0.26bc | 0.04bc | 0.05b | 0.11bc |
| *Lm* | D | 0.81 | 1.09 | 1.17 | 1.20 | 0.48 | 0.93 | 0.09 |
|  | R | 0.00a | 0.02ab | 0.17b | 0.15ab | 0.06ab | 0.20b | 0.00a |
| *Lp* | D | 0.00a | 0.00a | 0.00a | 0.03a | 0.30b | 0.41bc | 2.12c |
|  | R | 0.00a | 0.00a | 0.00a | 0.00a | 0.04ab | 0.00a | 0.11b |
| *Lar* | D | 24.91a | 14.14b | 5.99c | 0.57d | 0.04e | 0.01e | 0.00e |
|  | R | 0.18a | 0.52a | 0.66a | 0.29a | 0.05b | 0.01b | 0.04b |
| *Ts* | D | 4.60a | 4.57a | 5.41a | 2.22b | 3.71ab | 3.77a | 0.13b |
|  | R | 0.12a | 0.25b | 0.35b | 0.11a | 0.02a | 0.09a | 0.00a |
| *Co* | D | 0.39a | 0.52ab | 1.00b | 0.51a | 0.34a | 0.99b | 0.04a |
|  | R | 0.00 | 0.04 | 0.05 | 0.12 | 0.07 | 0.02 | 0.00 |
| *Cn* | D | 0.00a | 0.00a | 0.01a | 0.00a | 0.12b | 0.09b | 1.88c |
|  | R | 0.00a | 0.00a | 0.00a | 0.00a | 0.07b | 0.00a | 0.04b |
| *Cc* | D | 25.45a | 12.67a | 5.25ab | 0.00b | 0.00ab | 0.00ab | 0.00ab |
|  | R | 23.05a | 0.00b | 0.00b | 0.00b | 0.00b | 0.00ab | 0.00ab |
| *Bm* | D | 0.10 | 0.00 | 0.20 | 0.00 | 0.00 | 0.00 | 0.00 |
|  | R | 0.00 | 0.00 | 0.27 | 0.00 | 0.00 | 0.00 | 0.00 |
| *Ab* | D | 6.35a | 3.03ab | 2.49ab | 1.71b | 1.07b | 0.00b | 0.00b |
|  | R | 1.30 | 1.70 | 3.02 | 2.20 | 1.33 | 0.10 | 0.00 |
| *Md* | D | 0.00 | 0.03 | 0.07 | 0.04 | 0.20 | 0.00 | 0.00 |
|  | R | 0.00a | 0.00a | 0.09ab | 0.31b | 0.00a | 0.00ab | 0.00ab |
| *Alay* | D | 0.00 | 0.70 | 0.36 | 0.07 | 0.00 | 0.10 | 0.00 |
| *Cm* | D | 8.20ab | 17.63b | 12.87ab | 9.24a | 25.80bc | 52.90c | 1.00a |
|  | R | 8.95ab | 13.93a | 14.02ab | 13.58ab | 15.47ab | 79.70c | 0.00b |
| *Alat* | D | 1.50 | 5.93 | 2.87 | 1.62 | 2.67 | 0.00 | 0.00 |
|  | R | 1.20 | 1.63 | 2.04 | 1.42 | 6.13 | 0.00 | 0.00 |
| *Cl* | D | 5.20a | 16.20ab | 8.40ab | 4.04a | 1.67a | 19.90b | 1.60a |
|  | R | 0.70a | 9.93b | 10.33b | 10.18b | 1.00a | 10.60b | 2.80ab |
| *Ss* | D | 0.10 | 0.03 | 0.20 | 0.02 | 0.27 | 0.10 | 0.00 |
| *Isp* | D | 0.00a | 0.00ab | 1.05b | 0.22ab | 0.00ab | 0.00ab | 0.00ab |
|  | R | 0.00a | 0.00a | 2.02ab | 0.31b | 0.00ab | 0.00ab | 11.2c |
| *Iq* | D | 0.00a | 3.4abc | 9.29c | 6.42bc | 0.00ab | 0.40abc | 0.00abc |
|  | R | 0.90a | 0.00a | 0.44a | 0.00b | 0.00ab | 0.00ab | 0.00ab |
| *Isa* | D | 0.00a | 0.47a | 53.58b | 10.87b | 10.47b | 5.10b | 0.00a |
|  | R | 0.00a | 0.00a | 0.36a | 0.00a | 0.00a | 0.00a | 2.40b |
| *Nv* | D | 0.05a | 0.30a | 1.78b | 1.69b | 1.40b | 0.50ab | 1.40b |
|  | R | 0.05a | 0.07a | 2.47b | 2.71b | 3.33b | 2.20b | 5.40b |
| *Sj* | D | 0.35a | 0.73a | 11.60b | 14.29b | 47.8b | 35.7b | 0.00a |
|  | R | 0.00a | 0.00a | 5.49b | 5.47b | 2.60b | 1.30b | 2.40b |
| *Cdj* | R | 0.90a | 0.00b | 0.44b | 0.00b | 0.00b | 0.00b | 0.00b |
| *Pcy* | D | 2.40ab | 6.93b | 2.04ab | 1.78ab | 0.00a | 0.00a | 0.00ab |
|  | R | 2.40ab | 6.40b | 3.20ab | 1.78ab | 0.00a | 0.00ab | 0.00ab |
| *Lan* | D | 0.00a | 0.53ab | 3.20b | 0.36ab | 0.00ab | 0.00ab | 0.00ab |
| *Isc* | D | 7.20ab | 2.67a | 35.78b | 6.40ab | 1.07a | 1.60a | 6.40ab |
|  | R | 9.60 | 5.33 | 21.53 | 6.40 | 1.07 | 0.00 | 9.60 |
| *Gch* | D | 0.00 | 0.00 | 2.62 | 0.36 | 0.00 | 0.00 | 0.00 |
|  | R | 2.40 | 0.00 | 2.62 | 0.36 | 0.00 | 0.00 | 0.00 |
| *Ip* | D | 0.80a | 6.40a | 35.20b | 45.51b | 16.00ab | 6.40ab | 19.20ab |
|  | R | 0.00a | 0.53a | 16.87bc | 23.11c | 3.20ab | 0.00a | 9.60abc |
| *Gco* | D | 2.40a | 1.07a | 3.78a | 3.91a | 2.13a | 3.20a | 51.20b |
|  | R | 0.00a | 0.53a | 1.45a | 2.84ab | 2.13ab | 8.00b | 137.60c |
| *Me* | D | 0.00 | 1.60 | 3.49 | 1.78 | 0.00 | 0.00 | 0.00 |
| *Pcr* | D | 8.00 | 1.07 | 4.36 | 6.40 | 4.27 | 1.60 | 0.00 |
|  | R | 6.40a | 0.53ab | 0.29b | 0.71ab | 4.27a | 3.20ab | 0.00b |
| *Cdi* | D | 9.60ab | 11.20a | 21.53b | 17.42ab | 21.33b | 27.20b | 3.20a |
|  | R | 20.00 | 16.00 | 19.49 | 13.16 | 18.13 | 28.80 | 6.40 |
| *Sp* | D | 1.60 | 1.07 | 2.91 | 1.07 | 0.00 | 0.00 | 3.20 |

S2. Mean soil salinity (± standard error) at different elevations, based on salinity data from each sampling site. Bars labeled with different lowercase letters are significantly different (P < 0.05).

S3. Spearman's rank correlation coefficients between pairs of mangrove plants and soil salinity based on species abundance data from each sampling site. Asterisks correspond to significance: *, α = 0.05. Species abbreviations: *Ac, Aegiceras corniculatum; Am, Avicennia marina; Ko, Kandelia obovata; Bg, Bruguiera gymnorhiza; Eag, Excoecaria agallocha; Lr, Lumnitzera racemose*.

| Specis | *Ac* | *Am* | *Ko* | *Bg* | *Eag* | *Lr* |
| --- | --- | --- | --- | --- | --- | --- |
| ρ | −0.058 | 0.347* | 0.026 | 0.181 | −0.075 | 0.106 |
| P value | 0.739 | 0.038 | 0.882 | 0.291 | 0.663 | 0.539 |
| n | 36 | 36 | 36 | 36 | 36 | 36 |
